# Benefits on pain and mental health of manual therapy for idiopathic scoliosis: A meta-analysis

**DOI:** 10.3389/fendo.2022.1038973

**Published:** 2022-12-07

**Authors:** Jun Ren, Lingjun Kong, Zhiwei Wu, Xin Zhou, Qian Huang, Tianxiang He, Min Fang

**Affiliations:** ^1^ Yueyang Hospital of Integrated Traditional Chinese and Western Medicine, Shanghai University of Traditional Chinese Medicine, Shanghai, China; ^2^ Institute of Tuina, Shanghai Institute of Traditional Chinese Medicine, Shanghai, China; ^3^ Department of Acupuncture and Tuina, Lianyungang Traditional Chinese Medicine Hospital Affiliated to Nanjing University of Chinese Medicine, Lianyungang, China; ^4^ Shuguang Hospital, Shanghai University of Traditional Chinese Medicine, Shanghai, China

**Keywords:** manual therapy, idiopathic scoliosis, complementary and alternative therapy, mental health, pain

## Abstract

**Background:**

Idiopathic scoliosis (IS) is a common spinal disorder. Although several studies have reported the benefits of manual therapy for patients with IS in improving pain, anxiety, depression, and spinal disorders, the efficacy of manual therapy in the management of IS remain controversial. Therefore, this review was conducted to assess effects of manual therapy in the management of IS, primarily on pain and mental health of the patients and secondarily on their spinal disorders.

**Methods:**

Six electronic databases were searched for randomized controlled trials of manual therapy in the management of IS. The methodological quality of the included studies was assessed using the Physiotherapy Evidence Database (PEDro) Scale. The meta-analysis was conducted depending on different outcomes and control therapies using Review Manager version 5.3 software.

**Results:**

Seventeen studies were included in the present review. The PEDro scores of the included studies ranged from 5-7 points. The aggregated results indicated that Tuina (a traditional Chinese manipulation technique) had valuable improvement effects on pain (standardized mean difference (SMD), 0.92; 95% confidence interval (CI), 0.59 to 1.25; *P*<0.00001), negative emotions (SMD, 0.82; 95% CI, 0.51 to 1.13; *P*<0.00001), and disability (SMD, 1.29; 95% CI, 0.39 to 2.19; *P*=0.005). For the radiographic outcomes including the Cobb angle and vertebral rotation, Tuina, especially when combined with other conservative therapies, showed potential complementary effects for patients with IS.

**Conclusions:**

Tuina, as a complementary and alternative therapy, should be considered for the effective management of patients with IS, especially for the improvement of their pain and mental health. More randomized controlled trials are recommended to validate the current evidence.

**Systematic review registration:**

https://www.crd.york.ac.uk/prospero/, identifier CRD42020165220.

## Introduction

Idiopathic scoliosis (IS), a common spinal disorder with at least a 10° lateral curve of the spine, affects approximately 2-3% of adolescents worldwide (1, 2). In China, the prevalence of IS is as high as 5.1% among more than 200 thousand adolescent students (3). Patients with IS suffer from muscle pain, depression, anxiety, functional impairments, and poor quality of life (2, 4). Therefore, patients with IS and their families usually bear substantial socioeconomic burden and distress owing to the spinal deformities and their accompanying comorbidities (5).

The management options for IS include surgery and complementary and alternative therapies, including bracing, scoliosis-specific exercises, and manual therapy (4). Most patients with mild to moderate IS (Cobb < 40°) prefer complementary and alternative therapies to prevent and treat scoliosis, especially to improve pain and mental health. These physical and mental abnormalities are usually the main clinical symptoms in patients with mild to moderate IS. Having a good physical and mental health is essential for fulfilling lives, realizing one’s full potential, and contributing to the society. The International Society on Scoliosis Orthopaedic and Rehabilitation Treatment (SOSORT) also recommends complementary and alternative therapies to improve back pain, negative emotions, quality of life, and disability (2).

Manual therapy, as a complementary and alternative therapy for IS, is a skilled hand manipulative technique, including massage, chiropractic, osteopathy, and Tuina (traditional Chinese manipulation). It is comprised of soft-tissue manipulations (such as massage) and joint manipulations. Soft-tissue manipulations could restore the range of spinal motion by relieving muscle spasm (6). Joint manipulations (such as chiropractic) involve a manual procedure directed thrust to move a joint past the physiological range of motion, without exceeding the anatomical limit, which could improve stiffness of the spinal joints by adjusting the stress of intervertebral joints surface (7). Manual therapy could intervene the transcription and translation of inflammation-related genes through miRNAs to improve neuroinflammation and alleviate neuropathic pain (8). Although several trials have reported that manual therapy had benefits in improving negative emotions and spinal pain in patients with IS, the efficacy of manual therapy in the management of IS remains controversial (9, 10). In the previous review (11), there was no data synthesis in the meta-analysis due to the small number of eligible studies (12–14). Therefore, there is no positive recommendation for manual therapy in the treatment of IS in the SOSORT guidelines due to the lack of evidence-based results (2). In recent years, however, some randomized controlled trials (RCTs) were conducted to assess the efficacy of manual therapy in the treatment of IS (15–18). Particularly in China, Tuina is used in the management of IS by practitioners. Tuina comprise soft-tissue manipulations and joint manipulations. Compared to its role in correcting spinal abnormalities, it showed better potential effects in improving the pain and mental health of patients with IS.

Consequently, the current systematic review was conducted to examine the benefits of manual therapy in the treatment of IS. The Scoliosis Research Society (SRS) and SOSORT recommended that complementary and alternative therapies should focus primarily on the pain and mental health, and secondarily on spinal disorders (such as Cobb angle and vertebral rotation) in patients with IS (19). Therefore, the systematic review and meta-analysis was performed to evaluate the benefits of manual therapy in the management of IS, primarily in improving pain and mental health and secondarily in improving the spinal disorders.

## Methods

### Search strategy

A computerized literature search was conducted using the following electronic databases from their inceptions to January 2022: PubMed, Cochrane Library, EMBASE, CNKI, Weipu Database, and Wanfang Data. The key terms were scoliosis, manual therapy, chiropractic, massage, mobilization, osteopathy, myofascial release, spinal manipulation, Tuina, and shiatsu. Additionally, a manual search was conducted at the library of the Shanghai University of Traditional Chinese Medicine. Literatures were also obtained from the reference lists of related reviews. No restrictions were conducted for the language or publication status.

### Study selection

The criteria of the eligible studies were: (1) study design: RCTs as study design, (2) participants diagnosed with IS without any limitations on their gender or nationality, (3) intervention is manual therapy, such as Tuina, massage, chiropractic, mobilization, myofascial release, and shiatsu. The control interventions were any treatments without manual therapy, (4) outcomes are pain evaluated by any valid scale including visual analogue scale (VAS), mental health evaluated by any valid scale including self-rating depression scale (SDS) and self-rating anxiety scale (SAS), disability evaluated by any valid scale including oswestry disability index (ODI), and quality of life evaluated by any valid scale including scoliosis research society-22 (SRS-22), and spinal disorders (Cobb angle and vertebral rotation). Two reviewers independently selected studies. In case of persistent disagreement, a third reviewer will adjudicate and resolve the conflict.

Studies that included scoliosis patients undergoing combined orthopedic surgery were excluded. Likewise, studies involving IS patients with congenital spinal deformities or secondary to spinal organic diseases such as tumor, trauma, tuberculosis and other causes were also excluded.

### Data extraction

Data were independently extracted by two reviewers. The extracted information included the basic study information (first author, year, and country), participant information (sample size, mean age, Cobb angle, and main curve location), main outcomes, and intervention information. If disagreements persist, consensus will be adjudicated by the third reviewer.

### Quality assessment

Quality assessment of the included studies was conducted independently by the two reviewers using the Physiotherapy Evidence Database (PEDro) scale, which is an available evaluation method for methodological quality of randomized trials of physiotherapy interventions (20, 21). The PEDro scale contains the assessment of criteria regarding eligibility, randomised allocation of participants, concealed allocation, comparison of baselines, blinding (subjects, therapists, assessors), adequate follow-up, intention-to-treat, between-group comparisons, and point estimates and variability data. By scoring each of the last ten criteria, the quality of a paper ranges from low (0 point) to high (10 points), and the cut-off value for high-quality RCTs (6 points). In case of persistent disagreement, a third reviewer will adjudicate and resolve the conflict.

### Data synthesis and analysis

For the continuous data, standardized mean difference (SMD) and 95% confidence interval (CI) were used to conduct a meta-analysis using Review Manager version 5.3 software (the Cochrane Collaboration, Oxford, UK). We used a random-effects model for the better heterogeneity in the meta-analysis. The heterogeneity was assessed using the Cochran Q statistic (P value < 0.05 was considered statistically significant) and *I^2^* (*I^2^* > 75% considerable heterogeneity). Sensitivity analyses were also performed to investigate the impact of individual studies on heterogeneity measures. Subgroup analyses were performed depending on outcomes and control interventions. A comparison adjustment funnel plot was used to test the publication bias, when the number of studies included was larger than 10.

## Results

### Search and selection

**Figure 1:**
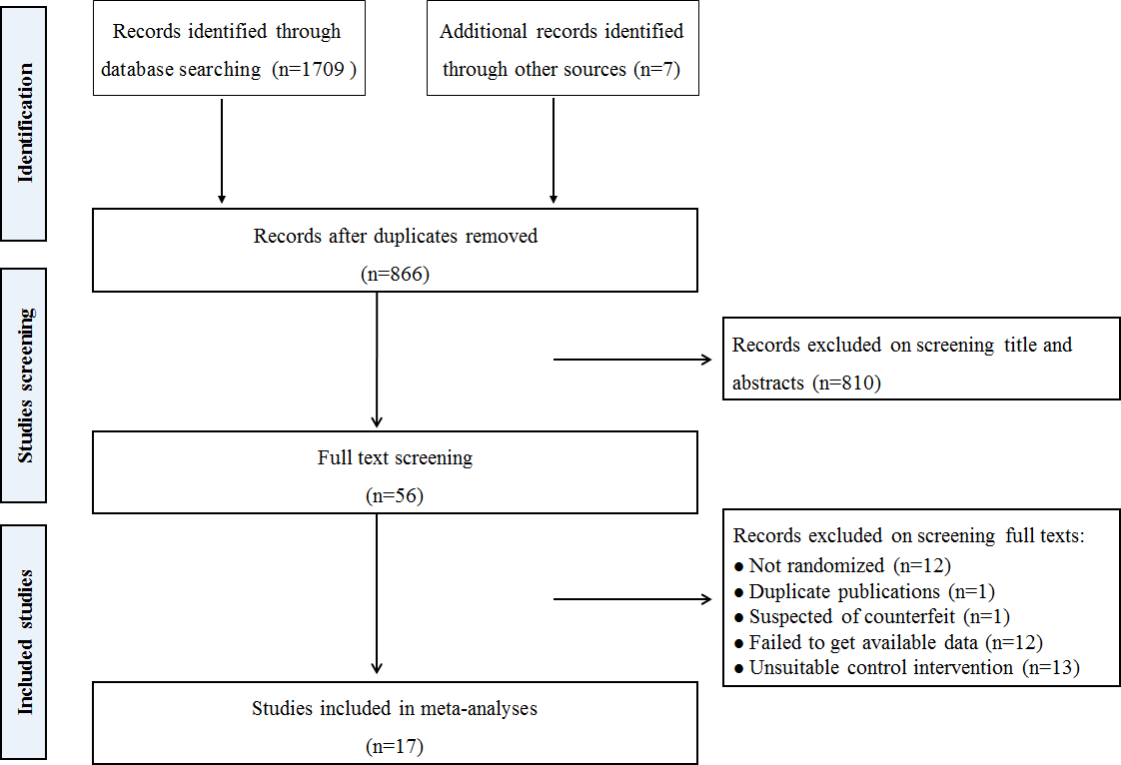
Study selection process.

A literature search identified 1716 records. 866 potential studies were found after removing the duplications. After screening titles and abstracts, 810 studies were excluded, and the remaining 56 full texts were screened for inclusion. After detailed full-text screening, 39 studies were excluded. Finally, 17 RCTs were included in the current review (15–18, 22–34). The detailed process of search and selection is presented in **Figure 1**.

### Characteristics of the included studies

Seventeen included studies conducted between 2003 and 2019 in China, USA, and Switzerland, which included a total of 956 participants with the mean Cobb angle 24.47° ± 5.26°. Manual therapy included Tuina, chiropractic, and osteopathic interventions. Ten studies used only manual therapy (Tuina or osteopathic) in the management of IS (15, 16, 18, 22, 26, 27, 31–34). Other studies combined manual therapy and other conservative interventions including bracing, exercise, traction, transcutaneous electrical stimulation, and medication. The control interventions included sham treatment, observation, bracing, traction, electroacupuncture, exercise and medication. The duration of the manual therapy ranged from 3-96 weeks. The time of each intervention ranged from 10-45 min. Two studies assessed the long-term effects of manual therapy for IS after the 3 (26) and 12 months (29). The characteristics of the included studies are summarized in **Table 1**.

**Table 1:** Randomized controlled trials of manual therapy for idiopathic scoliosis.

Study	Sample size	Mean age (year)	Cobbdegrees	Follow-up	Curve location*	Main outcomes	TCMintervention	Control group intervention
Liu,2003,China (22)	11 11 13 10	9-16	27.50±6.15 26.45±6.35 26.08±4.91 25.63±4.79	—	NR	Cobb, AVR	Tuina (120 sessions, 240 days)	Electroacupuncture (20 min, 120 sessions, 240 days) Tuinn plus electroacupuncture Observation
Quan,2006,China(23)	32 32	11.98±1.92 11.87±2.02	20.00±7.91 20.13±8.04	—	8,20,4 7,22,3	Cobb	Tuina (25 min, 24 sessions, 12 weeks) plus traction (15 min, 84 sessions, 12 weeks)	Brace (Boston) (22h/day, 12 weeks) plus traction (15 min, 84 sessions, 12 weeks)
Rowe,2006,USA(24)	2 3 1	14.50 13 16	21.50 29 18	—	0,1,1 2,1,0 0,0,1	Cobb, QOF	Chiropractic (32 sessions, 24 weeks) plus observation/brace	Observation/brace Sham chiropractic (32 sessions, 24 weeks) plus observation /brace
Hasler, 2010, Switzerland(15)	10 10	16.50 14.70	27.10 31.50	—	5,4,1 4,2,4	Spine flexibility, Trunk morphology	Osteopathic (3 sessions, 5 weeks)	Observation
Wan,2011,China(25)	20 20	14±3	28±11 27±10	—	NR	Cobb	Tuina (30 min/day, 12 weeks) plus traction (30 min/day, 12 weeks) and transcutaneous electrical stimulation and exercise	Traction (30 min/day, 12 weeks) plus transcutaneous electrical stimulation and exercise
Zhang,2011,China(26)	20 20	12.80±1.58 12.67±1.67	30.97±5.00 29.56±5.78	3 months	NR	Cobb, SRS-22, AVR	Tuina (30-45 min, 45 sessions, 12 weeks) plus brace (8 h/day, 12 weeks)	Brace (8 h/day, 12 weeks)
Wang,2014,China(27)	50 50	10~20	10~40	—	NR	Cobb	Tuina (35 sessions, 5 weeks)	Traction (20 min /35 sessions, 5 weeks)
Tian,2015,China(28)	20 18	66.63±7.73 63.51±6.61	17.87±6.23 18.01±5.67	—	NR	Cobb, VAS, ODI	Tuina (30 min, 36 sessions)	Celecoxib (200 mg/bid, 36 days) plus eperisone (50 mg/tid, 36 days)
Yang,2015,China(18)	42 42	11-23 11-22	32.20 31.80	—	NR	Cobb	Tuina (6 weeks)	Brace (23 h/day, 6 weeks) plus exercise (30-60 min/day, 6 weeks)
Lin,2016,China(29)	21 20	12.80±2.10 12.30±2.50	28.30±3.60 27.80±3.80	12 months	NR	Cobb	Tuina (12 sessions, 12 weeks) plus exercise (20-30 min, 6 sessions, 12 weeks)	Exercise (20-30 min, 6 sessions, 12 weeks)
Sun,2016,China(30)	30 30	61.80±6.90 63.60±4.80	18.10±6.40 18.90±5.80	—	NR	Cobb, VAS, ODI	Tuina (30 min, 10 sessions, 3 weeks) plus eperisone (50 mg/tid, 3 weeks)	Eperisone (50 mg/tid, 3 weeks)
Huang,2017,China(31)	20 20	9-15	20.54±5.21 21.51±5.09	—	NR	Cobb	Tuina (20 sessions, 20 days)	Brace (22 h/day, 20 days)
Zhao,2017,China(32)	30 30	64.80±4.90 63.70±4.60	16.83±4.26 16.54±4.32	—	NR	Cobb, VAS, ODI	Tuina (30 min/day, 36 days)	Celecoxib (200 mg/time, 2 time/day, 36 days) plus eperisone (50 mg/time, 3 time/day, 36 days)
Chen,2018,China(33)	41 41	NR	29.35±5.23 28.32±6.02	—	NR	Cobb, VAS	Tuina (24 weeks)	Brace (Cheneau) (24 weeks)
Li,2018,China(34)	40 40	11.68±1.69 12.23±2.07	21.85±2.97 22.53±3.19	—	NR	Cobb, EMG, SDS, SAS	Tuina (10-15 min/session, 90 sessions, 96 weeks)	Brace (Milwaukee) (22 h/day, 96 weeks)
Luo,2018,China(17)	37 39	12.68±1.53 12.18±1.59	18.43±6.50 20.87±9.69	—	NR	Cobb	Tuina (30 min, 36 sessions, 12 weeks) plus exercise (36 sessions, 12 weeks)	Exercise (36 sessions, 12 weeks)
Li,2019,China(16)	40 40	11.27±3.02	32.75±8.49 31.75±7.87	—	21,36,23	Cobb, Vertebral rotation	Tuina (8 sessions, 4 weeks)	Brace (23 h/day, 4 weeks) plus exercise (30-60 min/day, 4 weeks)

*Main curve location: participants with thoracic curve, thoracolumbar curve, and lumbar curve.

QOF, Quality of life; VAS, Visual analogue scale; NR, Not reported; SDS=Self-rating depression scale; SAS, Self-rating anxiety scale; ODI, Oswestry disability index; SRS-22, Scoliosis research society-22; AVR, apical vertebral rotation.

### Methodological quality

Most studies (88%) of manual therapy for IS, ranging from 5-7 on the PEDro scores, were rated as high quality with the exceeded cut-off PEDro score of 6. The most obvious bias was related to blinding and concealed allocations. Only one study performed concealed allocation (15), and two studies employed blinded assessors (15, 24). Furthermore, an intention-to-treat analysis was not used in three included studies (15, 33, 34). The other items in the included studies were scored as positive. Detailed scores are listed in **Table 2**.

**Table 2:** PEDro scale of quality for the included trials.

Study	Eligibility criteria	Random allocation	Concealed allocation	Similar at baseline	Subjects blinded	Therapists blinded	Assessors blinded	<15% dropouts	Intention-to-treat analysis	Between-group comparisons	Point measures and variability data	Total
Liu,2003 (22)	1	1	0	1	0	0	0	1	1	1	1	6
Quan,2006 (23)	1	1	0	1	0	0	0	1	1	1	1	6
Rowe,2006 (24)	1	1	0	0	0	0	1	1	1	1	1	6
Hasler, 2010 (15)	1	1	1	1	0	0	1	1	0	1	1	7
Wan,2011 (25)	1	1	0	1	0	0	0	1	1	1	1	6
Zhang, 2011 (26)	1	1	0	1	0	0	0	1	1	1	1	6
Wang,2014 (27)	1	1	0	1	0	0	0	1	1	1	1	6
Tian,2015 (28)	1	1	0	1	0	0	0	1	1	1	1	6
Yang,2015 (18)	1	1	0	1	0	0	0	1	1	1	1	6
Lin,2016 (29)	1	1	0	1	0	0	0	1	1	1	1	6
Sun,2016 (30)	1	1	0	1	0	0	0	1	1	1	1	6
Huang, 2017 (31)	1	1	0	1	0	0	0	1	1	1	1	6
Zhao,2017 (32)	1	1	0	1	0	0	0	1	1	1	1	6
Chen,2018 (33)	1	1	0	0	0	0	0	1	0	1	1	5
Li,2018 (34)	1	1	0	1	0	0	0	1	0	1	1	5
Luo, 2018 (17)	1	1	0	1	0	0	0	1	1	1	1	6
Li, 2019 (16)	1	1	0	1	0	0	0	1	1	1	1	6

Criteria (2–11) were used to calculate the total PEDro score. Each criterion was scored as either 1 or 0 according to whether the criteria was met or not, respectively.

### The effects on patient-centered outcomes

#### Pain

**Figure 2:**
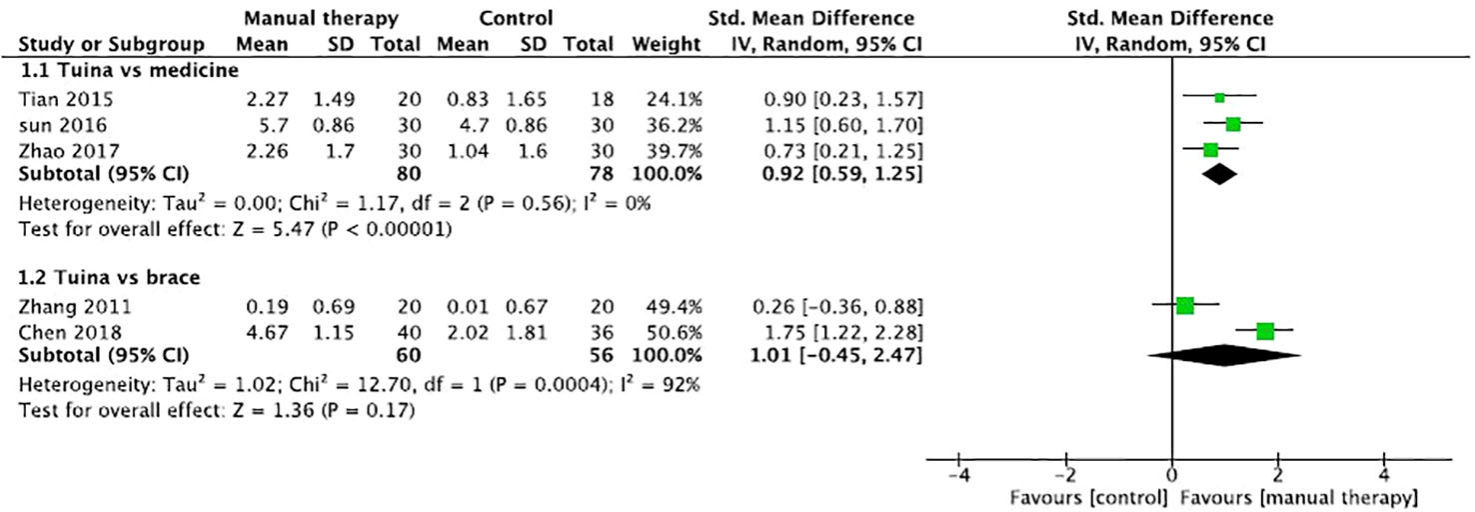
Forest plot of the effect of manual therapy for idiopathic scoliosis in pain.

The aggregated results of three studies reported the beneficial effects of Tuina in relieving pain (SMD, 0.92; 95% CI, 0.59 to 1.25; P < 0.00001; **Figure 2**) with low heterogeneity (*Q*=1.17, *I* ^2 =^ 0%; P=0.56) in patients with IS in comparison with those of medicines, including celecoxib and eperisone (28, 30, 32). Two studies assessed the effects of Tuina in relieving pain in comparison with those of braces (26, 33). However, the aggregated results failed to show that Tuina was more effective than braces in relieving pain (SMD, 1.01; 95% CI, -0.45 to 2.47; P = 0.17; **Figure 2**) with high heterogeneity (*Q*=12.70, *I* ^2 =^ 92%; P=0.0004).

#### Mental health

**Figure 3:**
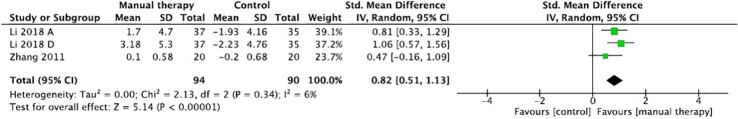
Forest plot of the effect of manual therapy for idiopathic scoliosis in mental health.

Two studies reported the effect of Tuina on depression or anxiety of patients with IS (26, 34). The aggregated result indicated that Tuina showed better effects in alleviating negative emotions including depression and anxiety (SMD, 0.82; 95% CI, 0.51 to 1.13; P<0.00001, **Figure 3**) with low heterogeneity (*Q*=2.13, *I* ^2 =^ 6%; P=0.34) compared with brace.

#### Disability

**Figure 4:**
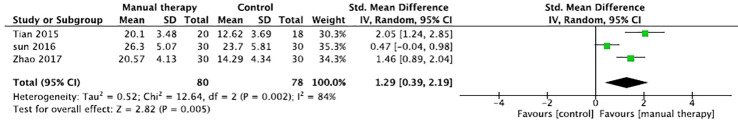
Forest plot of the effect of manual therapy for idiopathic scoliosis in disability.

Three studies reported the effect of Tuina in improving lumbar disability of patients with IS using the Oswestry disability index (28, 30, 32). The results of meta-analysis indicated that Tuina had beneficial effects in improving the disability (SMD, 1.29; 95% CI, 0.39 to 2.19; P=0.005, **Figure 4**) with high heterogeneity (*Q*=12.64, *I* ^2 =^ 84%; P=0.002) compared with medicine. In sensitivity analysis, exclusion of sun 2016 from the meta-analysis resulted in a bigger SMD of 1.68 (95% CI 1.13 to 2.24) and reduced heterogeneity (*Q*=1.34, I^2 =^ 25%, P=0.25).

#### Quality of life

In the included studies, only one reported the effect on quality of life of Tuina plus bracing for IS patients compared with bracing alone (26). The result showed that Tuina plus bracing have more benefits on domains of function/activity and pain of SRS-22 compared with bracing alone.

### The effects on radiographic outcomes

#### Cobb angles

**Figure 5:**
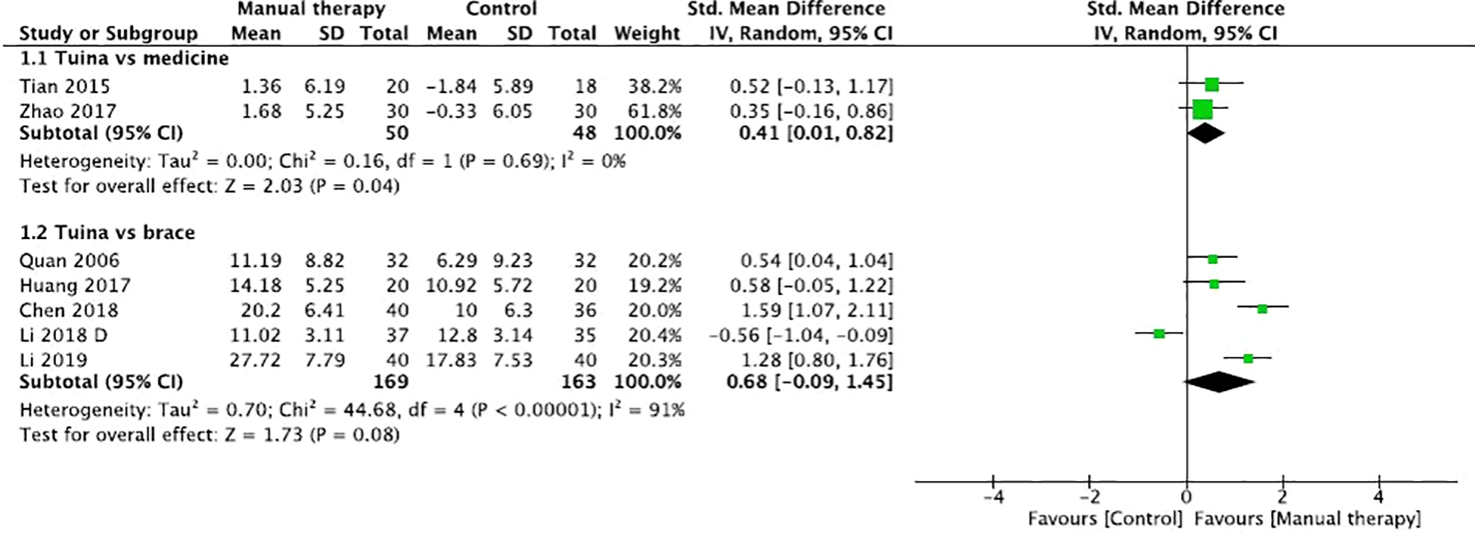
Forest plot of the effect of manual therapy for idiopathic scoliosis in Cobb angles.

Two studies reported the effects of Tuina in comparison with those of medicine in the management of Cobb angles in patients with IS (28, 32). The results of meta-analysis indicated that Tuina had better benefits than medicine in Cobb angles (SMD, 0.41; 95% CI, 0.01 to 0.82; P=0.04, **Figure 5**) with low heterogeneity (*Q*=0.16, *I* ^2 =^ 0%; P=0.69). However, five trials assessed the effect of Tuina compared with bracing (16, 23, 31, 33, 34). The aggregated result of the meta-analysis did not support better improvements of Tuina than bracing (SMD, 0.68; 95% CI, -0.09 to 1.45; P=0.08, **Figure 5**) with high heterogeneity (*Q*=44.68, *I* ^2 =^ 91%; P<0.0001). The exclusion of included studies from the analysis had minimal impact on the total estimate or on heterogeneity measures by sensitivity analysis. Wang et al. reported that Tuina had better benefits in Cobb angle of patients with IS compared with traction (27).

**Figure 6:**
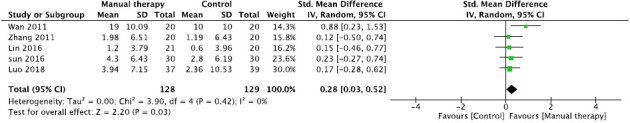
Forest plot of the effect of manual therapy plus other conservative therapies for idiopathic scoliosis in Cobb angles.

To achieve better efficacy, patients with IS usually prefer to combine manual therapy with other conservative therapies. In the included studies, the combination therapies included Tuina plus exercises, medicine, bracing, traction and transcutaneous electrical stimulation, and chiropractic plus observation/brace. The aggregated results indicated that Tuina plus other conservative therapies showed better complementary effects in improving the Cobb angles in patients with IS (SMD, 0.28; 95% CI, 0.03 to 0.52; P=0.03, **Figure 6**) (17, 25, 26, 29, 30) with low heterogeneity (*Q*=3.90, *I* ^2 =^ 0%; P=0.42). Rowe et al. reported no difference in the Cobb angles between patients tread with chiropractic plus bracing/observation, bracing/observation, and sham chiropractic, but this pilot study confirmed the strength of the existing protocols with amendments for use in a full RCT (24).

#### Vertebral rotation

**Figure 7:**
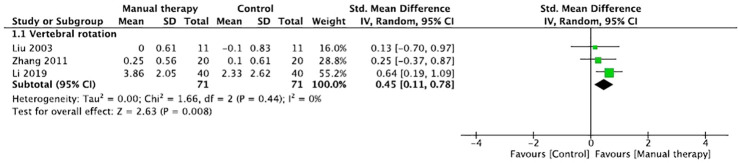
Forest plot of the effect of manual therapy for idiopathic scoliosis in vertebral rotation.

Three trials assessed the effects of Tuina on vertebral rotation in patients with IS (16, 22, 26). The aggregated results supported that Tuina showed more benefits in improving vertebral rotation (SMD, 0.45; 95% CI, 0.11 to 0.78; P=0.008, **Figure 7**) with low heterogeneity (*Q*=1.66, *I* ^2 =^ 0%; P=0.44). However, Hasler et al. reported that osteopathic treatment did not show better therapeutic effects on rib hump, plumb line, and sagittal profile of the spine than those by observation (15).

### The follow-up effects

Zhang et al. reported that manual therapy plus the brace showed better improvements in vertebral rotation, pain, and negative emotions than those by bracing alone, but no significant difference in Cobb angles was observed after 3 months of follow-up (26). Lin et al. reported that, after 12 months of follow-up, manual therapy plus exercise showed a better decreasing trend in the Cobb angles than that by exercise alone (29).

### Publication bias

The risk of publication bias was not assessed, because fewer than 10 studies were included in the meta-analysis. It means that the test power is too low to distinguish chance from real asymmetry (35).

## Discussion

To the best of our knowledge, the current study is the first systematic review with data synthesis of RCTs on manual therapy for IS. Subgroup analyses were conducted based on the different outcomes and control interventions. In terms of the patient-centered outcomes of the patients with IS, the current systematic review demonstrated that Tuina has valuable benefits in relieving pain, alleviating negative emotions, and improving lumbar disability. In the management of the Cobb angles, Tuina only showed better improvements than those by medicine, but Tuina plus other conservative therapies showed better effects in improving the Cobb angles in patients with IS than those by the conservative treatments alone. Tuina also has beneficial complementary effects on vertebral rotation in patients with IS. However, there is little evidence regarding follow-up effects of manual therapy for patients with IS.

Given that pain and negative emotions may be the chief reason for which patients with IS seek treatments, many complementary and alternative therapies may hold value even when they cannot produce radiographic changes of spinal deformity (36). For complementary and alternative therapies, the SOSORT and SRS also recommend that clinical outcomes (such as negative emotion, pain, and disability) relevant to patients with IS should be the primary focus (19). In the current review and meta-analysis, manual therapy, especially Tuina manipulation, showed valuable effects on pain and mental health, including depression and anxiety, which are important for IS treatment. This is usually the main reason why adults with IS visit clinics. In addition, the improvements in depression and anxiety might be beneficial for the long-term adherence to complementary and alternative treatments, and then contribute to halting the curve progression in patients with immature IS (37). These potential benefits should be assessed in the further high-quality clinical trials.

Most of the included studies focused on radiographic outcomes of patients with IS. In our review, Tuina showed beneficial improvements in the management of the Cobb angles and vertebral rotation in patients with IS. The current finding is interesting but insufficient to produce a reliable conclusion/recommendation. The progression of scoliosis differs significantly among the various Risser stages. However, few studies have reported the baseline Risser stage. The initial Risser stage is important for the research on patients with immature IS. For example, a stable Cobb angle from Risser 0 to Risser 3 may have better therapeutic benefits than a 6°curve correction during Risser 4 (13). Furthermore, the included studies did not report the number of patients with Cobb change of 6°or more, which was an improved standard in Cobb angles by the SOSORT and SRS. Several studies have reported that mean changes in the Cobb angles are less than 6°before and after the intervention (22, 28, 32). In addition, a minimum of 5-year follow-up period for adults with IS was recommended by the SOSORT criteria (13). There is little evidence of long-term effects of manual therapy on the Cobb angles. However, for the combined interventions, manual therapy showed beneficial complementary effects in halting/improving the curve progression. Therefore, the current results are interesting for future research.

## Limitation

The current study has a few limitations. The reviewers did their best to locate all eligible studies, but the distorting effects of publication and location bias on systematic reviews cannot be avoided. There were some flaws in the blinding methods used in the included studies. Although it was difficult to blind patients and therapists to manual therapy, blinded assessors should compensate for these flaws. Concealed allocation also should be performed to improve the study quality. In the meta-analysis, most studies used Tuina for IS. However, few studies included other manual therapies such as chiropractic, osteopathy, and massage. Moreover, the related studies were mostly conducted in China, and there were limitations in the regional distribution of the trials. Finally, few adverse events were reported in the included studies.

## Conclusions

The current review shows valuable evidence in support of Tuina for IS in terms of improving pain and mental health, and disability. The SOSORT and SRS also recommended that clinical studies should focus primarily on patient-centered outcomes, which are more important for IS, and secondarily on the radiographic outcomes. In radiographic outcomes, including the Cobb angle and vertebral rotation, Tuina, especially Tuina plus other conservative therapy including bracing and exercise, showed potential complementary effects for patients with IS. Therefore, manual therapy, especially Tuina, should be considered in management of patients with IS as a complementary and alternative therapy to improve their physical and mental well-being. More RCTs are recommended to validate the current findings.

## Data availability statement

The original contributions presented in the study are included in the article/supplementary material. Further inquiries can be directed to the corresponding author.

## Author contributions

Study design: LK, JR, and MF; Literature search: QH and JR; Data extract: XZ and JR; Methodological quality assess: XZ and LK; Data analysis: ZW, JR, and LK; Draft manuscript: MF, LK, ZW, and JR. The submitted version has been approved by all authors.
